# Editorial: CSF clearance in Alzheimer's disease and related dementias: exploring mechanisms and implications

**DOI:** 10.3389/fnagi.2025.1581223

**Published:** 2025-03-11

**Authors:** Yi Li

**Affiliations:** Weill Cornell Medicine, Cornell University, New York, NY, United States

**Keywords:** CSF clearance, Alzheimer's disease, glymphatic system, neurofluid dynamics, biomarkers, blood-brain barrier, sleep, neuroImage

Alzheimer's Disease (AD) and related dementias continue to rise globally, emphasizing the urgent need to elucidate the complex mechanisms underlying neurodegeneration. While the accumulation of amyloid-beta (Aβ) and tau proteins remains a core pathology, how effectively these proteins are cleared from the brain is emerging as a critical determinant of disease onset and progression. This Research Topic, *CSF Clearance in Alzheimer's Disease and Related Dementias: Exploring Mechanisms and Implications*, arrives at a pivotal moment in understanding neurofluid dynamics and its relevance to dementia.

As recent studies underscore, cerebrospinal fluid (CSF) and interstitial fluid (ISF) do far more than merely bathe neural tissues. The glymphatic system, a specialized network mediating CSF-ISF exchange, has reshaped our view of how the brain maintains metabolic homeostasis and eliminates waste. Disruptions in these delicate clearance pathways appear to fuel the accumulation of toxic protein aggregates, potentially exacerbating AD and other neurodegenerative conditions. This Research Topic assembles four key articles that highlight the multi-faceted nature of CSF clearance in AD and related dementias. Their diverse approaches—from exploring body position and nasal lymphatic outflow to applying machine learning on sleep data and examining blood-brain barrier (BBB) integrity—offer new avenues for both diagnosis and treatment. Below, we explore the major themes and contributions of this Research Topic.

## Exploring the mechanisms of CSF clearance

A cornerstone of this Research Topic is the exploration of the intricate mechanisms driving CSF clearance. We anticipate contributions that dissect the physiological processes involved—from CSF production at the choroid plexus, through its flow in the ventricular and subarachnoid spaces, to its interaction with ISF and ultimate drainage pathways. Understanding the driving forces behind CSF flow (e.g., cardiac pulsatility, respiration) and the roles of key cellular players like astrocytes and BBB permeability will be crucial.

Central to the study of neurodegeneration is the recognition that CSF flow and clearance pathways are dynamic. Body position, for instance, can alter the balance between cerebral blood flow and CSF circulation via gravity, cardiac pulsatility, and respiratory influences. In this Research Topic, Muccio et al. offer a comprehensive review of how posture (supine vs. upright) influences brain hemodynamics and CSF flow, highlighting the utility of advanced MRI techniques such as phase-contrast MRI for real-time assessment. Their work underscores how seemingly simple factors like body position can have significant implications for brain fluid physiology and overall brain health.

However, CSF outflow pathways extend beyond intracranial compartments. Building on the anatomical perspective, Phillips and Schwartz propose a novel hypothesis implicating nasal lymphatic obstruction as a potential etiology for AD. Based on nuclear medicine scan data, they suggest that metabolic dysregulation leads to parasympathetic overactivity and nasal turbinate vasodilation, thereby impeding CSF drainage through nasal lymphatics and facilitating waste accumulation central to AD pathology. This view aligns with prior PET research (J Nucl Med, 2017) indicating that the nose can serve as a viable drainage route—a concept that might inform future AD interventions (de Leon et al., 2017).

## Advanced imaging methodologies: illuminating neurofluid dynamics

The ability to visualize and quantify neurofluid dynamics *in vivo* is paramount for advancing our understanding of CSF clearance in both health and disease. This Research Topic is particularly enriched by studies leveraging nuclear medicine and Magnetic Resonance Imaging (MRI) techniques. Muccio et al.'s review highlights how phase-contrast MRI can capture hemodynamic and hydrodynamic changes in response to posture. Furthermore, recent research by Olivier Baledent's team expertly employs real-time phase-contrast MRI to elucidate the mechanisms behind CSF oscillations, demonstrating and quantifying the significant contributions of both breathing and cardiac cycles to cerebral arterio-venous blood flow dynamics, which in turn drive CSF movement (Liu et al., 2024). Beyond these techniques, other MRI based imaging markers also gained attention. DTI-ALPS quantifies water diffusion along perivascular spaces (PVS), aiming to capture the efficiency of CSF/interstitial fluid (ISF) dynamic in the glymphatic system (Taoka et al., 2017). By measuring the reduction of anisotropy of diffusion in directions orthogonal to fluid pathways, researchers can infer the impairment of perivascular channels. Meanwhile, the Brain Parenchymal CSF Fraction (pCSFF) map, derived from multi-echo T2 relaxometry, quantifies the fraction of CSF-like water volume within each MRI voxel of the brain parenchyma (Zhou et al., 2024). This approach offers novel insight into perivascular spaces, overcoming the challenge of MRI voxel size limitations by enabling assessment of glymphatic-related fluid accumulation across all types of PVS. Moreover, elevated pCSFF may reflect CSF/ISF stagnation and subsequent PVS enlargement. It serves as a new biomarker of glymphatic fluid accumulation or stagnation, potentially followed by the enlargement of PVS. Beyond DTI-ALPS and CSFF, other advanced MRI techniques like Dynamic DWI of PVS (Wen et al., 2022) and intrathecal MRI tracer injection (Eide and Ringstad, 2015) have also been recently published, further expanding the toolkit for studying the glymphatic system. The innovative use of nuclear medicine imaging is also brought to the forefront by Phillips and Schwartz, who utilize whole-body blood pool scans using radiotracers to observe nasal turbinate vasodilation, forming the basis of their novel AD etiology hypothesis. Combining advanced MRI and nuclear medicine methods offers a more holistic perspective, capturing both structural and functional aspects of fluid dynamics and extending the investigation to molecular or tracer-based assessments. By applying these advanced imaging methodologies synergistically, we stand poised to significantly advance our understanding of neurofluid interactions and revolutionize how we diagnose and treat disorders associated with compromised CSF clearance.

## Advanced methods for identifying CSF clearance dysfunction

Impairments in CSF clearance can manifest as altered biomarkers and subtle clinical changes early in the disease process. Two articles in this Research Topic explore innovative strategies for detecting these dysfunctions and improving diagnostic precision:

Gaeta et al. investigate the relationship between sleep disturbances, CSF clearance dysfunction, and Alzheimer's disease (AD) by applying a machine learning (ML) approach to predict CSF biomarkers (Aβ42, p-tau, t-tau) non-invasively. Their study builds on the established link between sleep quality, glymphatic function, and Aβ clearance, emphasizing how slow-wave sleep (SWS) facilitates neurofluid dynamics and waste removal. By analyzing polysomnography (PSG) features and clinical variables, they propose that sleep assessment via PSG could serve as an indirect indicator of CSF clearance efficiency and AD risk, potentially offering a way to reduce the need for invasive lumbar punctures. Their work explores how PSG-derived sleep measures, which reflect sleep quality and architecture, correlate with CSF biomarker levels, leveraging non-invasive sleep data as a proxy for underlying AD-related processes linked to CSF clearance.

Li et al. examine the cerebrospinal fluid/serum albumin ratio (Qalb) as a marker of blood-brain barrier (BBB) dysfunction in Lewy Body Disease (LBD). Their systematic review and meta-analysis reveal that Qalb is significantly higher in LBD compared to AD, suggesting that greater BBB impairment may serve as a distinguishing factor between these neurodegenerative conditions.

Together, these studies highlight how emerging diagnostic techniques, from sleep-based monitoring (PSG) to blood-CSF biomarker ratios (Qalb), can help identify distinct pathways of CSF clearance dysfunction in different dementias. These findings underscore the importance of integrating physiological, biochemical, and machine learning-driven approaches to enhance early detection and disease differentiation.

## Clinical and pathological relevance, and therapeutic implications

Collectively, the four articles emphasize that CSF clearance is influenced by multiple, interacting factors—body position, nasal lymphatic routes, sleep-related processes, and BBB integrity. It's also important to recognize that vascular health plays a significant role, as healthy cerebral blood vessels are crucial for efficient perivascular CSF flow and overall clearance (Saeed et al., 2023; Wang et al., 2022). By embracing a systems-level view of neurofluid dynamics, researchers and clinicians can better understand why some individuals exhibit faster cognitive decline while others remain relatively resilient.

This Research Topic also hints that diminished CSF clearance efficiency may hasten or exacerbate hallmark AD pathologies (Aβ plaques, tau tangles) and contribute to other neurodegenerative conditions like LBD. Identifying specific clearance deficits—whether at the level of posture, nasal outflow, sleep disturbance, or BBB compromise—can thus guide both diagnostic and therapeutic approaches.

CSF clearance pathways represent promising targets for intervention. Phillips and Schwartz propose addressing nasal turbinate vasodilation to restore lymphatic outflow, while Muccio et al. raise the possibility of postural interventions to enhance CSF dynamics. Emerging non-invasive therapies are also showing promise. For example, transcranial focused ultrasound stimulation is being investigated for its ability to enhance CSF circulation and promote waste clearance, offering a potential non-surgical approach for conditions like NPH and potentially AD (Yoo et al., 2023). Furthermore, immunomodulatory strategies, such as low-dose Interleukin-2 treatment, are being explored for their capacity to reduce neuroinflammation and enhance amyloid-beta clearance, suggesting a potential link to improved CSF drainage (Low Dose Interleukin-2, 2025). These suggestions align with the broader notion that glymphatic function can be modulated through behavioral or physiological adjustments (e.g., sleep hygiene), and that further research may yield specialized pharmacological or device-based therapies. As imaging and biomarker technologies progress, so do opportunities to measure and optimize neurofluid homeostasis.

## Conclusion: a call to explore the neurofluid frontier

By bridging mechanistic insights, advanced imaging, and clinical applications, this Research Topic—*CSF Clearance in Alzheimer's Disease and Related Dementias: Exploring Mechanisms and Implications*—offers a multifaceted look at how neurofluid dynamics shape dementia risk and progression. From body posture and nasal lymphatics to machine learning-based biomarker prediction and BBB assessment, the collected works underscore the importance of CSF clearance as both a diagnostic marker and a therapeutic frontier. We invite the scientific community to build on these discoveries—combining physiological, imaging, and computational advances—to further unravel the complexities of brain clearance and pave the way for novel dementia interventions.
